# *Heliotropium* (Boraginaceae) in the Marquesas Islands (French Polynesia) with description of a new species

**DOI:** 10.3897/phytokeys.47.8767

**Published:** 2015-03-17

**Authors:** David H. Lorence, Warren L. Wagner

**Affiliations:** 1 National Tropical Botanical Garden, 3530 Papalina Road, Kalaheo, HI 96741-9599, USA; 2 Department of Botany, MRC-166, National Museum of Natural History, Smithsonian Institution, P.O. Box 37012, Washington, DC 20013-7012

**Keywords:** *Heliotropium*, Boraginaceae, Marquesas Islands, French Polynesia

## Abstract

During the preparation of the Vascular Flora of the Marquesas Islands a new endemic species of *Heliotropium* L. (Boraginaceae) has come to light and is described herein: *Heliotropium perlmanii* Lorence & W. L. Wagner. It is known only from the island of Eiao and appears most closely related to *Heliotropium marchionicum* Decne., also endemic to the Marquesas and known from Nuku Hiva. An amended description of *Heliotropium marchionicum* and key to separate the Marquesan species are given and their differences discussed.

## Introduction

The Flora of the Marquesas Islands project is a collaborative program primarily between the Smithsonian Institution and the National Tropical Botanical Garden intended to further knowledge of the flora of this remote archipelago. In 1997 the first publications of new species and revisions of genera with at least one endemic species were initiated (Florence and Lorence 1997; Wagner and Lorence 1997). Since that time a series of publications has enumerated and revised a number of genera (for summary see Lorence and Wagner 2011). This treatment of the Marquesas species of *Heliotropium* L. is one of the last precursor publications before finalizing the data in the online Flora of the Marquesas Islands website (Wagner and Lorence 2002–).

A number of recent studies utilizing both molecular and morphological analyses suggest that the traditional Boraginaceae s.l. should be split into a number of families (see Refulio-Rodriguez and Olmstead 2014 and other papers cited therein). One of the primary reasons for this is that the overall clade is comparable to other nearby clades in the phylogeny that are treated as orders in the classification (Gentianales, Lamiales, and Solanales). Therefore, the group is being restructured to be a series of families within an order Boraginales. This classification would elevate former subfamilies of Boraginaceae to the rank of family (i.e., Boraginaceae, Cordiaceae, Ehretiaceae, and Heliotropiaceae); keep Hydrophyllaceae at the rank of family, but it may need to be split into two families); and recognize two small families, Wellstediaceae (formerly Boraginaceae) and Codonaceae (formerly Hydrophyllaceae). Refulio-Rodriguez and Olmstead (2014) point out that there are still a number of issues to resolve in the phylogeny of Boraginales that will affect the final classification of the clade. One issue is that one of the monophyletic groups, tribe Nameae of the Hydrophyllaceae, has no currently available family name. Since the overall new classification of the Boraginales requires further study to fully resolve, including proposal of at least one additional family, it seems premature to adopt it yet. For this reason we here use Boraginaceae in the broad sense for purposes of this contribution to the Flora of the Marquesas Islands project.

*Heliotropium* (Boraginaceae subfam. Heliotropoideae, or Heliotropiaceae of many authors) consists of 280 to 350 species of herbs, shrubs, lianas and small trees from the temperate and warm regions of the world, mostly in arid zones, with the greatest diversity in the New World (Diane et al. 2002; Luebert et al. 2011; Mabberley 2008; Wagner and Lorence 2002–). Molecular results using ITS1 demonstrated strong support for the Old World species of *Heliotropium* *s. str.*, but there are no clear morphological characters separating them from their New World sister clade (Diane et al. 2002). The systematics of this group remains highly controversial due to the scarcity of informative reproductive characters, i.e. floral and fruit morphology, and variability in leaf morphology. Since Pacific species were not included in analyses by either Diane et al. (2002) or Luebert et al. (2011), putative origin and affinities of the Marquesan species are unclear and further investigations are necessary to demonstrate their precise relationships.

In the Marquesas Islands (SE Polynesia) only a single native species, *Heliotropium marchionicum* Decne. has been previously recorded (Brown 1935, Drake del Castillo 1893), the type of which was collected at an unknown locality on Nuku Hiva island by Le Bastard. Study of *Heliotropium* collections for preparation of the Vascular Flora of the Marquesas Islands has revealed that the collections from Eiao differ from *Heliotropium marchionicum* in a number of significant, non-overlapping morphological features including branching of the stems, indument, phyllotaxis, characters of the flowers, including the annular stigma overtopped by a sterile, conically elongated stigmatic column, and fruits (see key below). For this reason we recognize the collections from Eiao as a new species, *Heliotropium perlmanii*. Risk evaluation for determination of conservation status was inferred using IUCN criteria for endangerment (IUCN 2001) based on best available information on suitable habitat and threats, primarily from personal observations by Jean-François Butaud (pers. comm. 2014).

## Systematics

### Key to Marquesas species of *Heliotropium*

**Table:** 

1	Stems virgately branched, with two subequal lateral branches developing adjacent to inflorescence; indument of trichomes 0.1–0.2 mm long; leaves opposite, blade with secondary veins obscure; calyx lobes unequal, 1–2 larger, ovate, 1–1.5 × 0.6–1 mm, 3–43 smaller, narrowly ovate to oblong, 1 × 0.3–0.4 mm; corolla 2 mm, tube 1–1.5 mm, lobes 0.5–0.7 × 0.5–0.7 mm; ovary glabrous except for ring of trichomes 0.2–0.3 mm long surrounding base of style; fruits 1 × 1.6 mm	***Heliotropium perlmanii***
–	Stems sympodially branched, with usually only a single lateral branch developing adjacent to inflorescence; indument of trichomes 0.2–0.5 mm long; leaves subopposite to alternate, blade with secondary veins visible; calyx lobes subequal, 1.7–2.2 × 0.7–1.2 mm; corolla 2.6–3.2 mm, tube 2.0–2.2 mm, lobes 1.2–1.7 × 0.8–1.3 mm mm; ovary densely strigillose; fruits 1.5–2 × 2 mm	***Heliotropium marchionicum***

### Heliotropium perlmanii

Taxon classificationPlantaeBoraginalesBoraginaceae

Lorence & W. L. Wagner sp. nov.

urn:lsid:ipni.org:names:77145753-1

#### Type.

**Figure 1.**
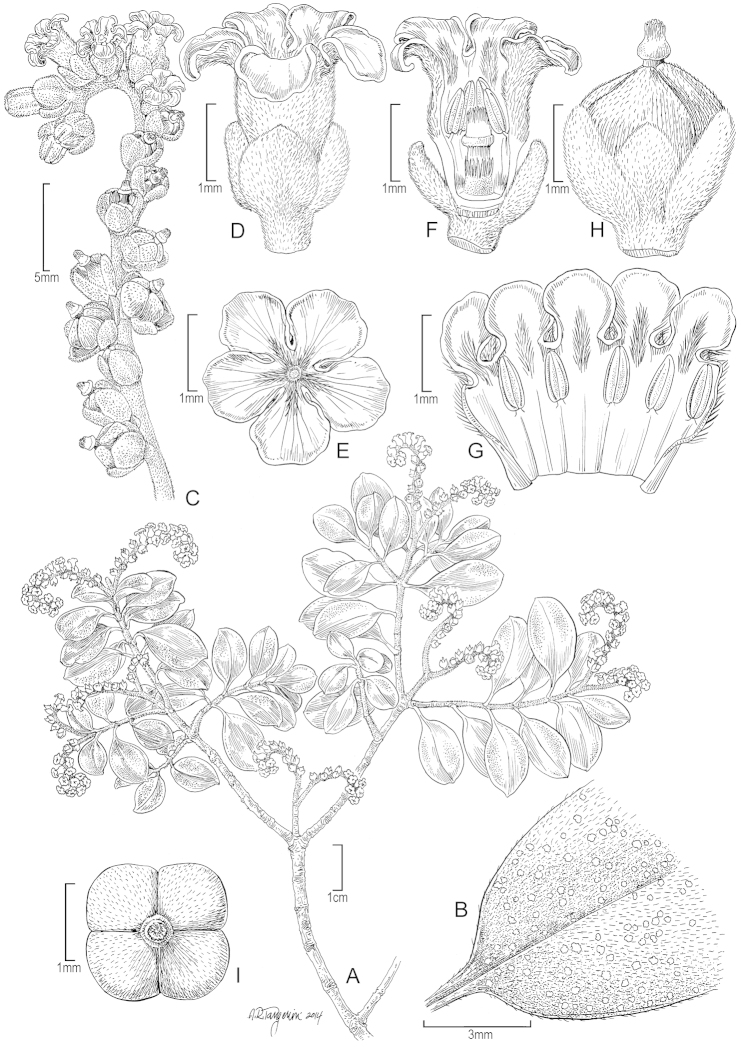
*Heliotropium perlmanii* Lorence & WL Wagner **A** Habit **B** Upper leaf surface, **C** Inflorecence **D** Flower, lateral view **E** Corolla, face view **F** Flower, longitudinal section showing stamens and gynoecium **G** Corolla, sectioned to show stamens and indument, **H** Fruit and calyx, lateral view **I** Fruit showing 4 carpels. All figures drawn from Perlman & Florence 10052 (US) and photos from Falaise Est Eiao, 11 March 2007 courtesy of J-F Butaud.

Marquesas Islands. Eiao, north side of large valley which is south of Vaittuha Valley, Opituha Valley. Sea cliffs, with Heliotropium, Dodonaea, Cordia lutea. Shrubs 1-2 ft. tall; flower; leaves smaller than Nuku Hiva plants; not silvery, 1050 ft [320 m], 7 Jul 1988, S. Perlman & J. Florence 10052 (Holotype PTBG 009229; Isotypes BISH, F, MO, P, PAP, US). Figure 1.

#### Description.

**Shrubs** 30–60 cm tall, stems decumbent, virgately branched, with two subequal lateral branches developing adjacent to inflorescence; leafy stems 0.8–1.5 mm in diam., terete, brown, moderately shortly strigillose-canescent with white ascendant trichomes 0.1–0.2 mm long; older stems with peeling brown bark. **Leaves** opposite, blade elliptic to broadly elliptic or obovate-elliptic, 0.8–1.8 × 0.3–1.0 cm, apex obtuse to rounded, or occasionally truncate, usually apiculate, base acutely cuneate, sides slightly attenuate and decurrent, subcoriaceous to coriaceous and brown when dry, bright green when fresh, both surfaces moderately shortly strigillose with appressed trichomes 0.1–0.2 mm, pustular, venation obscure, 1–2 (–3) pairs secondary veins arising near base, petiole 2–4 mm long, 0.4–0.5 mm in diam., shortly strigillose. **Inflorescences** terminal, scorpioid-cymose, forked 1(–2) times, axes densely shortly strigillose-canescent like the stems, 2–4 cm long, peduncle 0.5–0.8 cm long, primary axes 1.5–3 cm long. **Flowers** sessile to subsessile, 18–23 per axis, calyx lobes 5, unequal, 1–2 larger, ovate, 1–1.5 × 0.6–1 mm, 3–4 smaller, narrowly ovate to oblong, 1 × 0.3–0.4 mm, densely strigillose toward base, corolla shortly funnelform, 2 mm long, tube 1-1.5 mm long, externally densely villose-strigillose, internally slightly villosulous in throat, lobes 5, subcircular, 0.5–0.7 × 0.5–0.7 mm, margin crisped, stamens 5, attached midway in tube, basifixed, anthers ellipsoid, 0.5–0.6 mm long, apiculate; ovary cylindric-ovoid, glabrous except for ring of trichomes 0.2–0.3 mm long surrounding base of style, style terminal, 0.3 mm long, stigmatic column 0.3–0.4 mm long, cylindrical-conical, apex strigillose, base annular. **Fruit** broadly ovoid, 1 × 1.6 mm, shortly strigillose, shallowly 4-lobed, dry, splitting into 4 wedge-shaped nutlets. **Nutlets** 1.3–1.5 × 1–1.1 mm, dorsally strigillose, ventrally glabrous, brown.

#### Distribution.

Marquesas Islands, known only from three collections made on Eiao.

#### Habitat.

Grows on windward sea cliffs, with *Dodonaea viscosa* Jacq., *Cordia lutea* Lam., and *Bidens beckiana* (F. Br.) Sherff.

#### Conservation status.

Endangered (EN): B1ab (i, ii, iii) + 2ab (i, ii, iii): B2: total area of occupancy less than 500 km² (ca. 47 km²). B1a, severely fragmented; B1b (1–iii), habitat quality continuing decline inferred. The suitable habitat for *Heliotropium perlmanii* on Eiao (40 km²) is indicated as an endangered environment, threatened by feral animals and invasive plants, thus reducing the extent of the suitable habitat. Eiao has populations of feral sheep, pigs, cats, and rats (J.-F. Butaud, pers. comm. 2013).

#### Etymology.

We are pleased to name this new species in recognition of rough-terrain botanist Steven P. Perlman (National Tropical Botanical Garden) in recognition of his contributions to our knowledge of the flora of the Pacific region. Steve collected the type specimen and in his label data noted several differences from *Heliotropium marchionicum*.

#### Discussion.

Collections of this species were previously distributed as *Heliotropium marchionicum*, which differs by its non-virgate sympodial branching, more densely strigillose indument, larger leaves, and flowers about twice as large with corollas 2.6–3.2 mm long.

#### Specimens examined.

Marquesas Islands. Eiao: 20 September 1922, R.H. Beck & W.B. Jones 1537 (A, BISH); NW side of island, Vaituha Bay and summit ridge of island 400 m elevation, 1 August 1977, B.H. Gagné 1295 (BISH).

### Heliotropium marchionicum

Taxon classificationPlantaeBoraginalesBoraginaceae

Decne., Voy. Venus, Bot. [Alt.] 21. 1864.

#### Type.

**Figure 2.**
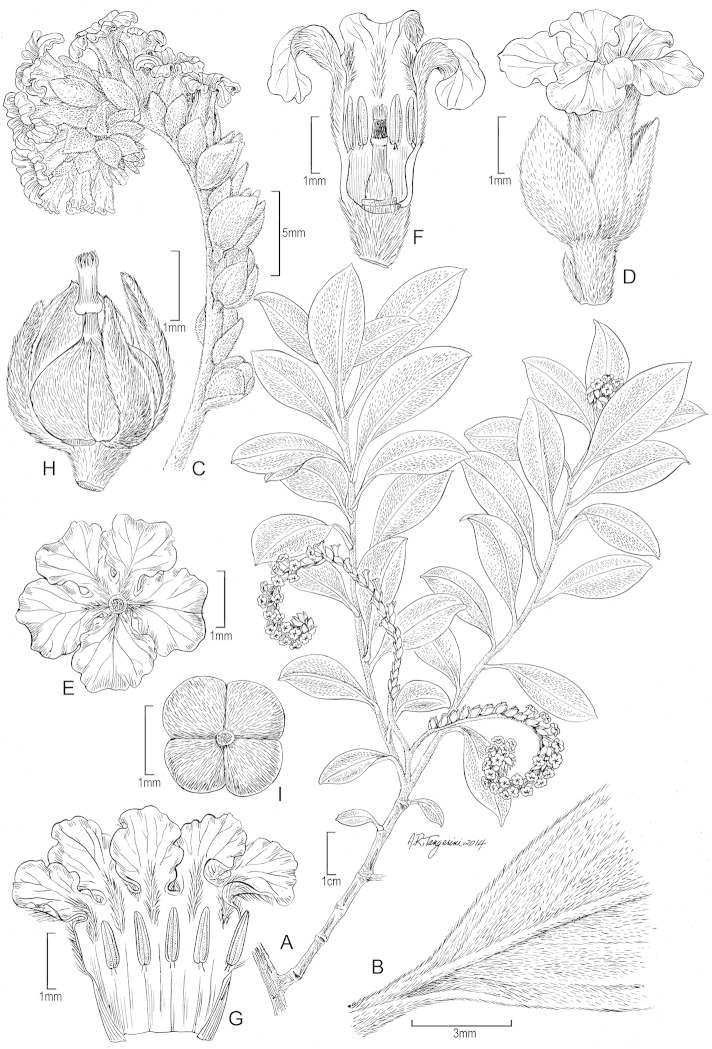
*Heliotropium marchionicum* Decne. **A** Habit **B** Upper Leaf surface **C** Inflorescence **D** Flower, lateral view **E** Corolla, face view **F** Flower, longitudinal section showing stamens and gynoecium **G** Corolla, sectioned to show stamens and indument **H** Fruit and calyx, lateral view **I** Fruit showing 4 carpels. Drawn from Perlman 10005 (US) and photos from Nuku Hiva, 24 February 2007 [**A**], Mercier 1847 (US) and photos from Nuku Hiva, 24 February 2007 courtesy of J-F Butaud [**B–I**].

Iles Marquises [Marquesas Islands], Noukahiva [Nuku Hiva], “toutemanou”, pl. herbacee sur le sommet du montagne, Le Bastard 76 (Holotype P, digital image!). Figure 2.

#### Description.

**Shrubs or suffrutescent perennials** 1–2 m tall, stems erect or decumbent, sympodially branched, with usually only a single lateral branch 30–120 cm long developing adjacent to inflorescence, terete, 1.5-3 mm diam., most parts densely silvery white strigillose with ascending white trichomes 0.2–0.5 mm long. **Leaves** subopposite to alternate, blade elliptic to narrowly elliptic or obovate-elliptic, 1–5 × 0.5–1.5 cm, apex acute, obtuse or rounded, often apiculate, base acute to narrowly cuneate, sometimes attenuate, chartaceous to subcoriaceous, both surfaces strigillose to densely white strigillose with appressed white trichomes 0.2–0.4 mm long, smooth or sometimes pustular, secondary veins 2–3 pairs arising in basal half of lamina; petiole 3–15 mm. **Inflorescences** terminal and later displaced by growth of one axillary bud, or sometimes leaf-opposed, scorpioid-cymose, densely white strigillose as for stems and leaves, 4–7 cm long, forked once, peduncle 1–2 cm long, primary branches 2.5–7 cm long, each with 17–35 flowers. **Flowers** sessile or subsessile, calyx lobes 5, densely white strigillose, free to the base, subequal, ovate to lanceolate, 1.7–2.2 × 0.7–1.2 mm, acute to acuminate; corolla shortly funnelform, 2.6–3.2 mm, tube 2.0–2.2 mm, externally strigillose except at base, internally with pubescent lines below the lobes, lobes 5, subcircular, 1.2-1.7 × 0.8–1.3 mm × 0.8 mm, margins crisped, dorsally strigillose medially; stamens 5, attached below middle of tube, basifixed, anthers linear-oblong, 0.6–0.7 mm long, glabrous, not connate; ovary ovoid, densely strigillose, 0.5 mm long, style terminal, 0.3–0.6 mm long, glabrous, stigmatic column 0.4–0.5 mm, cylindrical-conical, papillose, apex strigillose, base annular. **Fruit** broadly ovoid, 1.5–2.0 × 2.0 mm, shallowly 4-lobed, externally strigillose, dry, splitting into 4 wedge-shaped nutlets. **Nutlets** 1.4–1.6 × 0.8–1.0 mm, apiculate, dorsally densely strigillose, ventrally glabrous, dark brown.

#### Distribution.

Marquesas Islands, known only from Nuku Hiva.

#### Habitat.

This species usually occurs inland on basaltic cliffs and dry ridges, sometimes near waterfalls, in dry land forest with *Sapindus saponaria* L., *Cerbera manghas* L., and introduced invasive species including *Tecoma stans* (L.) Kunth and *Leucaena leucocephala* (Lam.) De Wit. The label on one collection notes it is a low elevation littoral plant (*Brown 542*, BISH).

#### Conservation status.

Proposed IUCN Red List Category Endangered (EN): B1ab (i, ii, iii) + 2ab (i, ii, iii): B2: total area of occupancy less than 500 km² (ca. 50 km²). B1a, severely fragmented; B1b (1–iii), habitat quality continuing decline inferred. The suitable habitat for *Heliotropium marchionicum* on Nuku Huka (ca. 340 km²) is indicated as an endangered environment, threatened by human activity (deforestation), feral animals, and invasive plants, thus reducing the extent of the suitable habitat.

#### Discussion.

*Heliotropium marchionicum* is apparently closely related to *H. permanii* but differs by the characters noted above. A single collection from Taiohae, Nuku Hiva (Florence 8394, BISH, CHR, K, NY, P, US) resembles *Heliotropium marchionicum* superficially but differs in having stems and petioles pilose with hairs to 1 mm long, inflorescence axis pilose, very small flowers (calyx lobes 1–1.1 mm long, corolla 1.1–1.3 mm long) and ribbed fruits 1.1–1.2 × 1.8 mm, covered with bulbous-tuberculate scales, splitting into 4 nutlets. Further collections are needed to determine whether it represents an undescribed taxon or alternatively a naturalized species. It closely resembles *H. angiospermum* Murr., native to North America and the Caribbean and was identified by M. Strong (US) as this species.

#### Specimens examined.

Marquesas Islands. Nuku Hiva: Hakaui, 20 July 1921, F.B.H. Brown 542 (BISH); Moyenne vallée de Hakaui, flanc droit, 125 m, latitude 08°54'S, longitude 140°10'W, 18 May 1984, J. Florence 6695 (BISH, P); Hakaui Valley, 107 m elevation, 26 June 1988, S. P. Perlman 10005 (AD, BISH, F, MO, MU, NY, OS, P, PAP, PTBG, US); Matatekouaehi Valley, about 2 miles in from coast, by 100 ft. waterfall, 1 July 1988, S. P. Perlman 10026 (BISH, PTBG, US); Taiohae, flanc gauche de la baie, S du CJA, 150 m, latitude 08°56’S, longitude 140°05’W, 26 Jul 1987, J. Florence 8394 (BISH, CHR, K, NY, P, US),W shore, 200 m elevation, 20 Oct 1922, W.B. Jones 1591 (BKL); slope on ridge, 18 Oct 1922, E.H. Quayle 1591 (A, BISH); without precise locality, 1841, R. Hinds s.n. (P), Mathias 96 (GH), 1847, M.P. Mercier s.n. (P, US).

## Supplementary Material

XML Treatment for Heliotropium perlmanii

XML Treatment for Heliotropium marchionicum

## References

[B1] Brown FBH (1935) Flora of Southeastern Polynesia. III. Dicotyledons. Bernice P. Bishop Mus. Bull. 130: 1–386.

[B2] Diane N Förther H Hilger HH (2002) A systematic analysis of *Heliotropium*, *Tournefortia*, and allied taxa of the Heliotropiaceae (Boraginales) based on ITS1 sequences and morphological data. Am. J. Bot. 89: 287–295. doi: 10.3732/ajb.89.2.287 21669738 10.3732/ajb.89.2.287

[B3] Drake del Castillo E (1893) Flore de la Polynésie française. Libraire de l’Académie de Médecine, Paris, 352 pp.

[B4] Florence J Lorence DH (1997) Introduction to the flora and vegetation of the Marquesan Archipelago. Allertonia 7: 226–237.

[B5] IUCN (2001) IUCN Red List categories (version 3.1). IUCN Species Survival Commission, Gland, Switzerland http://www.iucnredlist.org/info/categories_criteria2001

[B6] Luebert F Brokamp G Wen J Weigend M Hilger HH (2011) Phylogenetic relationships and morphological diversity in Neotropical *Heliotropium* (Heliotropiaceae). Taxon 60: 663–680.

[B7] Lorence DH Wagner WL (2011) Introduction to Botany of the Marquesas Islands: new taxa, combinations, and revisions. Phytokeys 4: 1–4. doi: 10.3897/phytokeys.4.1781 22171178 10.3897/phytokeys.4.1781 PMC3174443

[B8] Mabberley DJ (2008) Mabberley’s Plant-Book. Cambridge University Press, 1021 pp.

[B9] Refulio-Rodriguez NF Olmstead RG (2014) Phylogeny of Lamiidae. Am. J. Bot. 101: 287–299. doi: 10.3732/ajb.1300394 24509797 10.3732/ajb.1300394

[B10] Wagner WL Lorence DH (1997) Studies of Marquesan Vascular Plants: Introduction. Allertonia 7: 221–225.

[B11] Wagner WL Lorence DH (2002–) Flora of the Marquesas Islands website. http://botany.si.edu/pacificislandbiodiversity/marquesasflora/index.htm [accessed October 2013]

